# Expression of endocan and vascular endothelial growth factor in recurrent minor aphthous ulcers

**DOI:** 10.4317/jced.55695

**Published:** 2019-06-01

**Authors:** Enji A. Mahmoud, Wesam-Abdel Moneim, Olfat G. Shaker, Dalia M. Ghalwash

**Affiliations:** 1 Associate professor in Department of Oral Medicine and Periodontology, Faculty of dentistry, Cairo University; 2 Professor in Department of Biochemistry, Faculty of Medicine, Cairo University; 3 Associate Professor of Oral Medicine and Periodontology, Faculty of dentistry, The British University in Egypt (BUE), Cairo, Egypt

## Abstract

**Background:**

Recurrent aphthous ulcers (RAU) are common painful inflammatory lesions of the mucous lining of the mouth. Endocan, previously identified as endothelial cell specific molecule-1, is implicated as a vital player in the regulation of several inflammatory processes. A number of inflammatory cytokines and pro-angiogenic growth factors including VEGF upregulate endothelial cells synthesis and expression of endocan.

**Material and Methods:**

Clinical scores of pain and ulcer size as well as level of endocan and VEGF were determined in swaps from aphthous ulcer and contra lateral normal mucosa in 30 patients (nine males and twenty one females) with age ranging from 18 to 45 years and mean age is 31.5 years.

**Results:**

In the early days of ulcer development, ulcer showed statistically significantly higher mean endocan (8.2 ±5.3) and VEGF levels (1220.7 ±294.6) than control healthy mucosal site (1.1 ±0.5) and (518.6 ± 61.7) respectively. An increase in endocan is associated with an increase in pain score and vice versa. A statistically significant positive correlation were also found between endocan and VEGF levels.

**Conclusions:**

Endocan and VEGF are strongly associated with the destructive phase of minor aphthous ulcers especially Endocan which was positively correlated with pain severity.

** Key words:**Endocan, ESM-1, VEGF, Recurrent Apthous Ulcer.

## Introduction

Recurrent aphthous ulcers (RAU) is the most communal inflammatory ulcer affecting the oral mucosa (1) with the prevalence of 5 - 25 % in the overall population. The peak period of RAU occurs in the second decade and the first occurrence being in early childhood or even in later (2). The decreased occurrence of RAU in elderly may be due to age-dependent variations in the acquired and innate immune systems, designated as immunosenescence (3).

Based on the clinical presentation, three forms of RAU can be designated: minor, major or herpetiform aphthous ulcers. The minor type is the most common affecting about 80% of patients (1). It occurs as round or oval ulcers <1 mm in diameter affecting non-keratinized mucosa as the buccal and labial mucosa and floor of mouth with the floor covered by a grayish white pseudomembrane and is surrounded by a halo of erythema. The ulcers usually heal in 10–14 days with no scarring (4).

Thus far, the etiopathogenesis of this disease remains uncertain and seems to be multifactorial. The possible risk factors that could trigger the condition include: food allergies, vitamin deficiencies, genetic predisposition, bacterial and viral infections, systemic ailments (e.g., ulcerative colitis, Crohn’s disease, celiac disease, AIDS), hormonal disorders, amplified oxidative stress, mechanical irritation and stress (5-7).

The most accepted theory regarding the aetiology of RAU is that these predisposing factors or unknown antigenic stimulation triggers oral mucosal keratinocytes to transform into a target of the uncontrolled action of lymphocytes, neutrophils and monocytes resulting in liberation of acute inflammatory mediators, damage of oral mucosal cells, thus the appearance of an aphthous ulcer (8,9).

The acute inflammatory mediators liberated during the initiation of the ulcerative process that included cytokines for instance TNF-α, IFN-ϒ, IL-2 and IL-2, along with the migration of other lymphocytes, langerhans cells and neutrophils also causes an over expression of Vascular Cell Adhesion Molecules (VCAM), Intercellular Adhesion Molecules (ICAM-1) and Selectine E leading to more lymphocytic accumulation and invasion of the epithelium locally (10).

Vascular endothelial growth factor (VEGF) is a powerful endothelium-specific cytokine that effectively stimulates angiogenesis, inflammation, endothelium-dependent vasodilatation and amplified microvascular permeability (11). It is principally produced by vascular endothelial cells, macrophages and neutrophils. In inflammatory reactions its production is stimulated, and VEGF itself endorses the inflammatory processes. An increase in VEGF levels have been described in several inflammatory and autoimmune diseases (12,13). In their study, Arbiser *et al.* (14) reported elevation in VEGF levels in addition to its receptors in RAU and advocated that oral aphthous ulcerations might be highly angiogenic.

Endocan is a unique human endothelial cell- specific molecule (ESM). It was initially cloned from a human endothelial cell DNA library by Lassalle *et al.* (15). Endocan is secreted by epithelial cells of the renal distal tubules, vascular endothelial cells, bronchi and lung sub mucosal glands (16). A number of cytokines and growth factors regulate the expression of endocan by vascular endothelium. Binding of endocan to human leukocytes occurs via the integrin leukocyte function-associated antigen (LFA)-1 and hinders the interactions of LFA- 1/intercellular adhesion molecule (ICAM)-1 and, possibly, LFA-1-mediated leukocyte functions (17). Endocan expression has been reported to be intensely up-regulated via proangiogenic molecules as VEGF-A and VEGF-C which are known to be critical mediators of lymphangiogenesis, angiogenesis, and cancer progression (18).

To the best of our knowledge, there is no previous study investigating the association of endocan and VEGF in recurrent aphthous ulcers. Therefore, in the present study, we aimed to investigate the expression levels of Endocan and VEGF in minor aphthous ulcer lesions during both the active and the healing stages.

## Material and Methods

This study was conducted in Faculty of Dentistry, Cairo University after approval of the faculty Research Ethics Committee.

Thirty subjects (nine males and twenty one females) were recruited from outpatient clinic of Oral Medicine, and Periodontology Department, Faculty of Dentistry, Cairo University.

Inclusion criteria included patients having minor aphthous ulcers with no other specific pathology affecting the oral mucosa. The patients were systemically free according to Cornell medical index. The appearance of the ulcers should not be more than 3 days at the time of enrolment in this study.

Exclusion criteria included: patient with any systemic disease producing oral ulcerations as Bechets disease, ulcerative colitis, Reiter syndrome, haematological diseases, Cohns disease, allergic conditions, nutritional deficiencies, in addition to patients who have received any medical treatment in the past six months, pregnant or lactating mothers and smokers.

During the period of clinical study, the subjects had not to use any topical agents such as mouth wash and topical medication for treating aphthous ulcer and avoid taking spicy foods, sour drinks and coffee. Patients were informed of the nature and objectives of the study and they were educated about importance to come for the follow up visits till the end of the study. After that the enrolled patients signed a written informed consent.

Clinical evaluation of the patients:

1. Recording of pain score:

Assessment of pain was done via Visual Analogue Scale (VAS) that is comprised of a 10 cm line enclosing equidistant subdivisions of the following concomitant scores: randling.

Score 0 = no pain

Score 1 = pain with rough aggravation

Score 2 = pain with moderate aggravation

Score 3 = pain with slight aggravation

Score 4 = constant pain

Score 5 = severe pain

Patients were informed that aggravation referred to anything that may move or touch the ulcer area and also that pain associated with gentle touching of the lesion was greater than that of rough handling.

Pain scores were assessed 2 times; in the second and the tenth day of the ulceration period. Pain scores were plotted in a table and compared in the study site statistically.

2. Assessment of ulcer size as follows:

Under good lightening conditions, the ulcer area was dried carefully then a transparent plastic sheet was cut to facilitate its application directly on the ulcer. Then using a permanent water proof marker pen, the circumference of the ulcer was traced on the plastic sheet. The tracing was then placed on graph paper and the size of the ulcer was calculated in mms. Ulcer sizes were assessed 2 times; in the second and the tenth day of the ulceration period. The sizes of the lesions were plotted in a table and compared in the study site statistically.

Exfoliative cytology and preparation of smears:

Swabs were taken from the ulcer at the second and tenth days of the ulceration period as well as from the contralateral healthy mucosa to assess the level of Endocan and VEGF.

The sampling procedure was done as described previously by Spafford *et al.* (19). Aphthous ulcers were cleaned slowly with a cotton wetted by saline and then the ulcers were swabbed with a sterile cotton roll. This technique was applied by a stroke of the mucosa 3 to 5 times to collect the cell exfoliations. In addition, swabs from the contralateral clinically unaffected mucosa were obtained from all subjects as well to compare the level of Endocan and VEGF in the ulcer and the healthy mucosa.

After swabbing of the ulcer and mucosa by the cotton roll, the sample was allowed to dissolve in an eppendorff tube containing Phosphate Buffered Saline (PBS) solution. Samples were then stored at – 70O C until being used for laboratory analysis.

Assessment of Endocan and VEGF:

Endocan Assay:

Endocan was measured using ELISA kit supplied by EIAab, China Catalogue No: E2112h. This kit allows for the *in vitro* quantitative determination of human Endocan concentrations in serum, tissue homogenates and different biological fluids. The microtiter plate of the kit has been pre-coated with an endocan specific antibody. This is followed by addition of samples and standards to the microtiter plate wells with a biotin-conjugated polyclonal endocan specific antibody. Horseradish Peroxidase (HRP) is then added to and incubated in the microplate well. Then substrate solution (TMB) is added to all wells. The reaction is finished by the addition of a stop solution of sulphuric acid and the color intensity is measured by spectrophotometer at a wavelength of 450 nm ± 2 nm. The Endocan concentration in the samples is specified from the standard curve.

VEGF Assay:

The concentration of VEGF was determined by a quantitative sandwich enzyme immunoassay kit (R&D Systems, Minneapolis, MN.) according to manufacturer’s directions. Briefly, 100 ml assay diluent RD1W was added to each well. After dilution of samples, 100 ml sample was added per well. It was covered with the adhesive strip provided and incubated for 2 hours at room temperature. Aspiration and washing for each well was done and repeated twice for a total of three washes. A total of 200 ml VEGF conjugate was added to each well, covered with a new adhesive strip, and incubated at room temperature for 2 hours. Aspiration and washing was repeated. A total of 200 ml substrate solution was added to each well, protected from light, and incubated at room temperature for 25 minutes. Then, 50 ml stop solution was added to each well. The plate was gently tapped to confirm complete mixing. Assessment of optical density of the wells was done within 30 minutes via a microplate reader set to 450 nm. VEGF levels in samples were appraised via the standard curve plotted utilizing the optical density values with the standards. Samples and standards were assayed in duplicate as recommended by the manufacturer.

-Statistical Analysis

Data were presented as mean, standard deviation (SD), median and range values. Data were explored for normality by checking the data distribution, calculating the mean and median values and using Kolmogorov-Smirnov and Shapiro-Wilk tests. All data showed non-parametric distribution. The study is a split-mouth design; so Wilcoxon signed-rank test was used to study the changes after treatment as well as to compare between ulcer and control sides.

Spearman’s correlation coefficient was used to determine significant correlation between different variables.

The significance level was set at *P* ≤ 0.05. Statistical analysis was performed with IBM® SPSS® Statistics Version 20 for Windows.

## Results

The present study included a total of thirty subjects (nine males and twenty one females). The mean ± standard deviation (SD) values of age were 33.2 ± 10.2 years with a minimum of 18 years and a maximum of 45 years old.

Comparison of the different variables between the second day and the tenth day

Pain score and ulcer size:

The pain and ulcer size were recorded at the second day and again at the tenth day of ulcer period. Pain score decreased significantly from 3.6 ± (0.9) at the second day to 1.0 ± (0.7) at the tenth day with *p*-value <0.001*.

The same was observed with the ulcer size which was 35.1 ± (9.8) mm2 at the second day and significantly reduced to 11.6 ± (5.8) mm2 at the tenth day with p-value <0.001* ( Table 1).

**Table 1:**
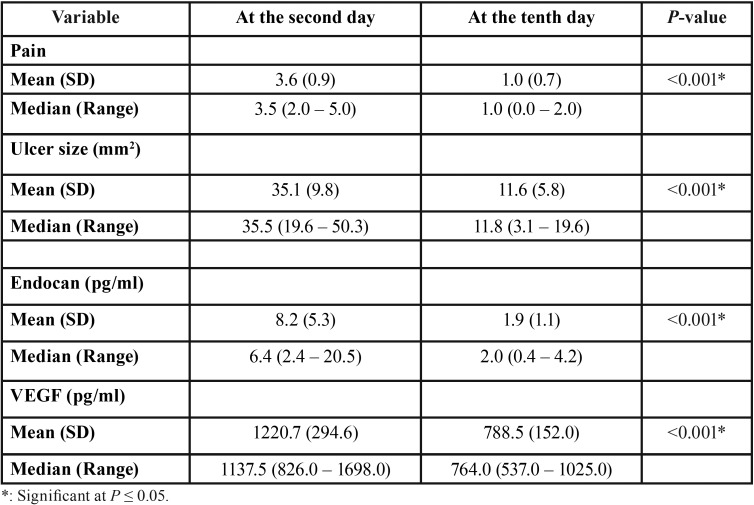
Descriptive statistics and results of Wilcoxon signed rank test for the changes in different variables.

Endocan and VEGF level:

At the second day of ulcer activity, the ulcer swabs showed statistically significantly higher mean endocan levels 8.2 ± (5.3) pg/ml than those at the tenth day 1.9 ± (1.1) pg/ml with *p*-value <0.001*. Similarly, the ulcer swabs showed statistically significantly higher mean VEGF levels at the second day of ulcer activity 1220.7 ± (294.6) pg/ml than those at the tenth day 788.5 ± (152.0) pg/ml with *p*-value <0.001* ( Table 1).

Comparison of Endocan and VEGF level between study and control sides

At the second day of ulcer activity, the ulcer swabs showed statistically significantly higher mean endocan and VEGF levels 8.2 ± (5.3) and 1220.7 ± (294.6) pg/ml respectively than those of the healthy mucosa 1.1 ± (0.5) and 518.6 ± (61.7) pg/ml respectively with *P*-value < 0.001 and < 0.001, respectively. Similarly, during healing at the tenth day; the ulcer swabs showed statistically significantly higher mean endocan and VEGF levels 1.9 ± (1.1) and 788.5 ± (152.0) pg/ml respectively than those of the healthy mucosal site 1.1 ± (0.5) and 518.6± (61.7) pg/ml respectively *P*-value = 0.009 and < 0.001, respectively ( Table 2).

**Table 2:**
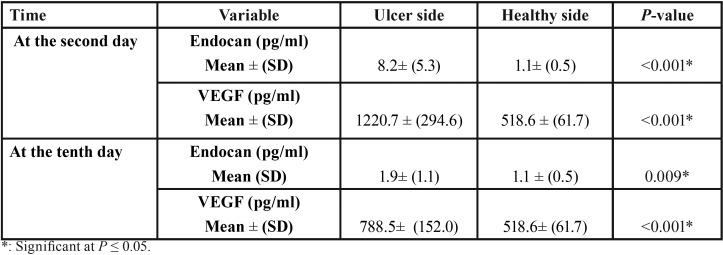
Descriptive statistics and results of Wilcoxon signed-rank test for comparison between study and control sides.

Correlation between different variables in the study side

**Figure 1:**
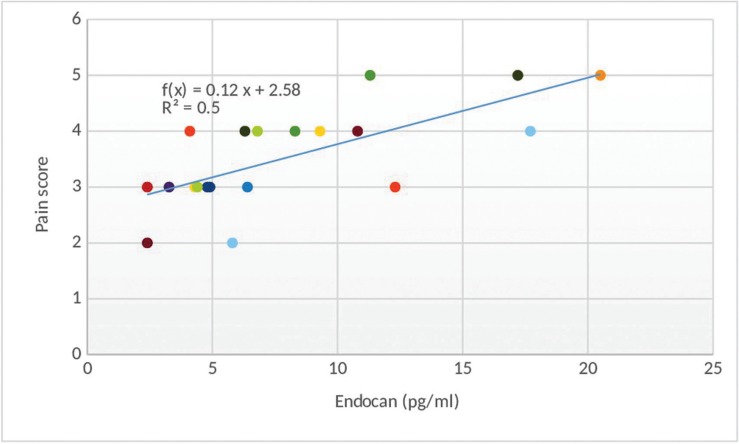
Scatter diagram representing direct correlation between endocan and pain scores at second day.

**Figure 2:**
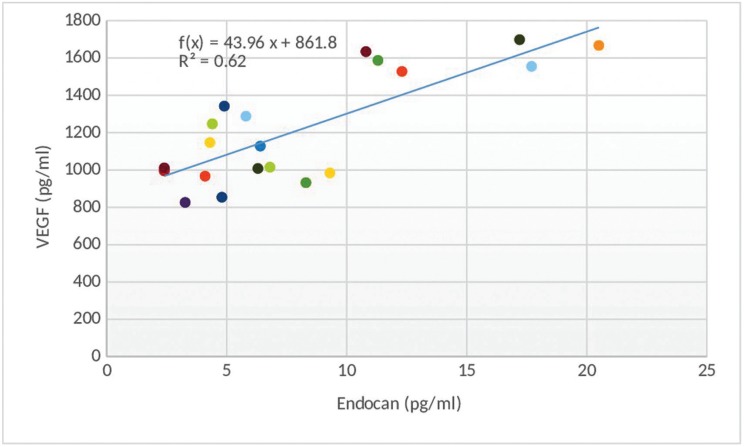
Scatter diagram representing direct correlation between Endocan and VEGF at second day.

At the second day, there was a statistically significant positive (direct) correlation between endocan and pain score; i.e. an elevation in endocan is associated with an elevation in pain score and vice versa (Fig. 1). There was also a statistically significant positive (direct) correlation between endocan and VEGF; i.e. an elevation in endocan is associated with an elevation in VEGF and vice versa (Fig. 2).

No statistically significant correlation was found between (endocan and ulcer size), (VEGF and pain score) as well as (VEGF and ulcer size).

At the tenth day, there was no statistically significant correlation between any of the different variables ( Table 3).

**Table 3:**
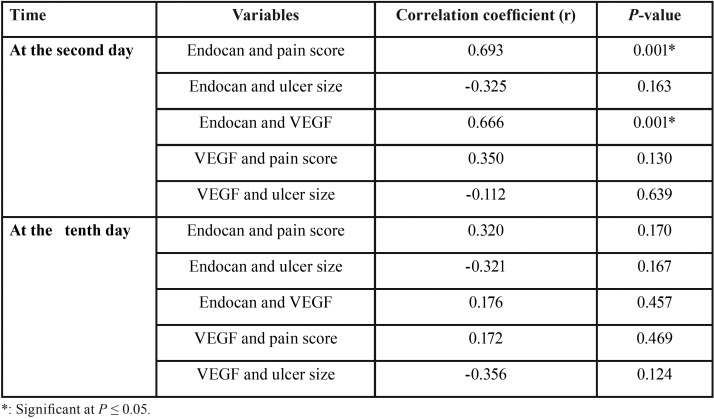
Results of Spearman’s correlation coefficient for the correlation between different variables.

## Discussion

Although the exact aetiology of RAU is unknown, several investigations have suggested immunodysregulation in this patients (2,20,21). Many studies also emphasized the role of the autoimmunity in the disease development. Disturbance of acquired as well as innate immune response may occur in patients with RAU demonstrated as elevated levels of the complement proteins, neutrophils hyper-reactivity, elevated amount of B lymphocytes and NK cells, in addition to disruption of the CD4/CD8 ratio and elevated amount of T cell receptor (TCR) and CD25 cells in peripheral circulation (11,22).

Endothelial cells are in charge of leukocytes attraction which are recruited early in inflammatory response (23). The Th1 immunologic response is suggested by many authors to have a crucial role in the progression of RAU (20,21,24). This was supported by Lewkowicz *et al.* (25) who reported a significant increase of Th1 cytokines in RAU patients in contrast to healthy subjects.

Vascular endothelial growth factor (VEGF) is produced by platelets and plays an imperative role in proteolytic enzyme secretion, proliferation of endothelial cell, and cell emigration via chemotaxis; in addition to its angiogenesis stimulating function (26).

Endocan, a soluble proteoglycan secreted by vascular endothelial cells and it is primarily regulated by various inflammatory cytokines as IL-1 and TNF-a. In addition to VEGF-A which is also implicated in the action of endocan. Moreover, some experimental studies proposed endocan as a strategic regulator of inflammatory disorders, cellular adhesion, and cancer development. Therefore, it is considered an endothelial immunoinflammatory marker (17,27).

To the best of our knowledge, the present research is the first to evaluate the role of endocan in the pathogenesis of recurrent aphthous ulcer.

The present research aimed to evaluate the pain severity, ulcer size, and cytological profile of both endocan and VEGF in RAU in the active phase at the second day and in the healing phase at the tenth day. Pain score as well as ulcer size were found to significantly decrease from the second day of ulcer development to the tenth day. Both findings are consistent with the self-limiting nature of RAU aphthous in which healing usually occurs within 10-14 days (2). Accordingly at day ten ulcers had already entered the healing phase with reduction in ulcer size and consequently pain score being a symptom of active destruction .

Since no previous studies evaluated endocan levels in oral exfoliative cytology specimens in aphthous ulcers, it is hard to compare our results directly with other researches. However, several studies reported a significant increase in serum level of endocan in other inflammatory disorders as septic shock (28), psoriasis vulgaris (29), cardiovascular events (17) and chronic kidney disease (30). Other studies reported that endocan level can be elevated in malignancies, however the regulation of its secretion and synthesis carried out via proinflammatory cytokines, as well as angiogenic factors like VEGF (31,32).

Moreover, endocan has been strongly associated with Behçet Disease which is an inflammatory condition characterized by more than 95% occurrence of oral aphthous like ulcers (33).

Findings of the present study showed that mean levels of endocan was statistically significantly higher in oral RAU lesions than control side either at second day or at day ten. Moreover, the ulcer swabs showed statistically significantly higher mean endocan levels at the second day of ulcer activity than those at the tenth day during the healing phase indicating the strong association of endocan with acute destructive phase of the disease.

Balta *et al.* (27) reported that endocan levels in serum of Behçet Disease patients were significantly higher than control and discovered that serum endocan levels directly correlated with erythrocyte sedimentation rate, C-reactive protein, and with disease activity. Moreover, endocan levels were greater in patients suffering from systemic manifestations, and they concluded that endocan may impact both endothelial function and the inflammatory process through the pathogenesis of Behçet Disease and attributed the elevated endocan levels found in those patients to the LFA-1/ICAM- 1 pathway inhibition; and stimulation of endocan expression by the raised VEGF plasma levels in Behçet Disease patients (34).

Additionally our results revealed a statistically significant positive (direct) correlation between endocan levels and pain score, where an increase in endocan level was associated with an elevation in pain score and vice versa indicating that endocan is more strongly associated with disease severity and active destructive phase of minor aphthous ulcers.

The higher level of endocan can be explained by the fact that many of cytokines and growth factors regulate its expression (17). Where it was found that TNF-α and IL- 1β and VEGF had been shown to prompt endocan expression (35). Since it has been reported that such cytokines and growth factors are directly involved and may be overexpressed during the destructive process of oral aphthous ulcer (2,10). Consequently this could explain the elevated endocan levels encountered in RAU lesions in the present study.

Another explanation is that endocan may be involved in regulating leukocyte extravasation at sites of inflammation, due to the importance of the ICAM-1/LFA-1 interactions for secure adhesion of monocytes and lymphocytes. In addition, endocan could modify the LFA-1/ICAM-1 co-stimulatory pathway on T cells and could modulate the balance of Th1/Th2 in the immune response (34). Such balance is disturbed favoring Th1 type immunologic response which is proposed by many authors to play the fundamental role in RAU development (21,22,25) reflecting a conceivable connection of endocan to the pathogenesis of RAU.

The present study also investigated the levels of VEGF in oral RAU due to its relation to endocan as endocan synthesis is enhanced during inflammatory processes, and VEGF is found to promote inflammatory reactions by mobilizing leukocytes (25). Furthermore, VEGF itself was reported to upregulate endocan expression (18). This link was endorsed by the results of the present study where a statistically significant positive (direct) correlation between endocan and VEGF was found during the dynamic phase of the disease as an increase in endocan level was associated with an increase in VEGF level and vice versa.

Morover, VEGF is considered as a potent angiogenic factor in the pathogenesis of vasculitis of aphthous ulcers which are characterized by great angiogenic action and a loss of the epithelial integrity in the oral mucosa (36). Hence, anti-angiogenic treatments as thalidomide have been proposed to endorse healing of oral ulcers and to control the frequency of recurrences (14). Moreover, VEGF induces vascular thrombosis and inflammation via stimulating endothelial cells causing the release of vasoactive substances (37).

While investigating VEGF levels in the present study mean levels of VEGF was statistically significantly higher in diseased side than control side either at second day or at day ten. Moreover, the ulcer swabs showed statistically significantly higher mean VEGF levels at the second day of ulcer activity than those at the tenth day during the healing phase. This evident association linking VEGF to the ulcerative process identified in our study is consistent with former studies associating the levels of VEGF with oral aphthous ulceration and with proof from other ulcer-related disorders as Behçet Disease where clinical proof advocates that VEGF could be directly involved in oral ulcers formation in Behçet Disease (11,14,37,38). Finally, an association between the genetic pathway of VEGF and the occurrence of oral ulcers have been recognized and validated the in Systemic Lupus Erythematosus (39).

Furthermore, topical steroidal and non-steroidal therapies that have been utilized effectively for the management of RAU are found to considerably affect the VEGF pathway genes expression designating that the VEGF pathway might be a significant mediator of the benefits of immunotherapeutic agents for oral ulcers.

## Conclusions

The results of the present investigation revealed a strong association of Endocan and VEGF levels with the destructive phase of RAU with Endocan levels being more directly correlated to pain scores. Furthermore a significant positive correlation between Endocan levels and VEGF levels was evident validating the interrelation between these two markers during the ulcerative process. Therefore, our findings suggest that the Endocan and VEGF interconnection along with their association with the phase of active destruction might offer new insights for a novel anti-inflammatory strategy of oral RAU that might allow more specific remedy while avoiding the detrimental side effects of existing therapies.
